# Improving the peer review of narrative literature reviews

**DOI:** 10.1186/s41073-016-0019-2

**Published:** 2016-09-04

**Authors:** Jennifer A. Byrne

**Affiliations:** 1 Molecular Oncology Laboratory, Children’s Cancer Research Unit, Kids Research Institute, The Children’s Hospital at Westmead, Locked Bag 4001, Westmead, 2145 NSW Australia; 2 The University of Sydney Discipline of Child and Adolescent Health, The Children’s Hospital at Westmead, Locked Bag 4001, Westmead, 2145 NSW Australia

**Keywords:** Peer review, Narrative literature review

## Abstract

As the size of the published scientific literature has increased exponentially over the past 30 years, review articles play an increasingly important role in helping researchers to make sense of original research results. Literature reviews can be broadly classified as either “systematic” or “narrative”. Narrative reviews may be broader in scope than systematic reviews, but have been criticised for lacking synthesis and rigour. The submission of more scientific manuscripts requires more researchers acting as peer reviewers, which requires adding greater numbers of new reviewers to the reviewing population over time. However, whereas there are many easily accessible guides for reviewers of primary research manuscripts, there are few similar resources to assist reviewers of narrative reviews. Here, I summarise why literature reviews are valued by their diverse readership and how peer reviewers with different levels of content expertise can improve the reliability and accessibility of narrative review articles. I then provide a number of recommendations for peer reviewers of narrative literature reviews, to improve the integrity of the scientific literature, while also ensuring that narrative review articles meet the needs of both expert and non-expert readers.

## Background

Over the past 30 years, the size of the published scientific literature has expanded exponentially [1]. While it has been argued that this rate of expansion is unsustainable [2], underlying factors such as greater numbers of scientists and scientific journals [3] are unlikely to change in the short term. The submission of more manuscripts for publication requires more peer reviewers, yet the current demand for capable, available manuscript reviewers is not being met [3]. This has serious adverse consequences for the validity of published research and overall trust in science [3].

Review articles help both experts and non-experts to make sense of the increasing volume of original publications [4, 5]. Busy clinicians have a particular reliance upon review articles, because of their constant need for reliable, up-to-date information, yet limited available time [6]. Literature reviews can also help other content experts such as researchers and policymakers to identify gaps in their own reading and knowledge. However, literature reviews are also sought by readers with little or no prior understanding of the reviewed topic, such as researchers seeking to rapidly triage results from high-throughput analyses and students for whom literature reviews can represent entry points into a new field. For the benefit of both expert and non-expert readers, it is essential that review articles accurately synthesise the relevant literature in a comprehensive, transparent and objective manner [7, 8].

Numbers of review articles are increasing in fields where this has been measured [4], as is the diversity of review types published [9, 10]. Although there are now many review sub-types that can be distinguished based upon the literature search, appraisal, synthesis and analysis methods used [9, 10], review articles can be broadly classified as either “systematic” or “narrative” [5, 11]. Systematic reviews take defined approaches to the identification and synthesis of study findings and include other review sub-types such as evidence maps [12]. The systematic review is considered to be the gold standard of evidence synthesis, but also carries the potential disadvantages of narrow scope [11], and requiring more time and resources to prepare and update [7]. Narrative reviews, also referred to as “traditional reviews” [5] and “literature reviews” [9], constitute the majority of review articles published in some fields [7]. Other review sub-types, such as rapid and scoping reviews also present information in a narrative format [9]. Narrative reviews have been criticised for rarely employing peer-reviewed methodologies, or duplicate curation of evidence [5], and for often failing to disclose study inclusion criteria [11]. Despite these limitations, narrative reviews remain frequent within the literature, as they offer breadth of literature coverage and flexibility to deal with evolving knowledge and concepts [11]. In this article, I will provide advice regarding the peer review of narrative reviews, and the advice presented aims to be broadly applicable. I will not attempt to provide advice regarding the peer review of systematic reviews [13, 14].

Given the broad readership of literature reviews, content and methodology experts as well as reviewers with less directly relevant expertise can play important roles in the peer-review process [15]. Peer reviewers with related content expertise are best placed to assess the reliability of the information presented, while other reviewers can ensure that this information remains accessible to readers with different levels of prior knowledge. However, whereas there are easily accessible guides for reviewers of primary research manuscripts [16, 17], there are few similar resources available for reviewers of literature reviews [15, 18]. This article therefore proposes a number of recommendations for peer reviewers (Table 1) to ensure that narrative literature review articles make the best possible contributions to their fields, while also meeting their readers’ often diverse needs.

**Table 1:** Summary of issues to consider during peer review of narrative literature reviews and their significance

Questions for peer reviewers	↑Scientific integrity	↑Information accessibility
Is the review article required/important?	√	√
Was the conduct of literature searches defined?	√	√
Were literature citations appropriate and balanced?	√	
Were original references cited?	√	√
Was information summarised correctly?	√	
Were studies critically evaluated?	√	√
Are there adequate tables/figures/diagrams?	√	√
Will the review help readers entering the field?		√
Does the review expand the body of knowledge?	√	√

### Ask whether the literature review justifies its place in the literature

Lower than expected ratios between numbers of original publications and review articles suggest excessive numbers of reviews in some fields, which may contribute to the very problem that review articles aim to solve [4]. With rapidly rising publication rates in many fields [2], even content-expert peer reviewers should check publication databases for similar and/or overlapping review articles as part of the peer-review process. Pre-empting such scrutiny, authors should clearly define the review’s scope and what it intends to achieve [8]. If there have been other recent reviews of the same or similar topics, the authors should explain how their manuscript is unique. This could be through combining literature from related fields, by updating existing reviews in light of new research evidence [8], or because published reviews may have been subject to bias. A clear definition of a review’s scope is a recognised tool to reduce evidence selection bias [19]. Review authors can also define their subject by referring to literature reviews of related topics that will not be explored in depth. These definitions and statements should form part of an overall narrative structure that helps readers to anticipate and understand the information presented [20].

### Ask whether the literature searches conducted were clearly defined

A criticism frequently levelled at traditional or narrative reviews is that they do not always state or follow rules regarding literature searches [5, 7, 11]. Providing evidence that comprehensive literature searches have been conducted, preferably according to pre-defined eligibility criteria [19], increases confidence that the review’s findings and conclusions are reliable, and have not been subject to selection bias. Ideally, any literature search choices made by the authors should be clearly stated, transparent and reproducible [11].

### Check for citation breadth and balance

Consider whether the authors have cited a comprehensive range of literature or whether they have tended to cite papers that support their own point of view. If there are important papers that have not been cited, suggest to the authors that these be added, and explain why. If only a limited number of articles can be cited due to the journal’s requirements, check that these studies are representative of those available.

### Where possible, verify that information has been summarised correctly

Many different types of citation errors can be identified in the research literature [21, 22], and these may occur regardless of the journal impact factor [22]. The increasing size and complexity of primary reports [3] also render data extraction and summary more challenging. Realistically, it is unlikely that individual peer reviewers will have detailed knowledge of any full review topic [19]. Nonetheless, if you are a content expert, take time to cross-reference at least some individual statements to citations, for the particular benefit of non-expert readers. If your level of expertise means that you are unable to verify the accuracy of particular sections of the review, you should indicate this to your editor. Peer reviewers can also ask about data extraction methods, if these were not described in the manuscript. Adopting systematic review practices, such as duplicate independent data extraction, or independent data extraction and validation, can reduce content errors and increase reliability [19].

### Check that original references have been cited

Authors sometimes incorrectly cite original studies, both in original manuscripts and reviews [23, 24]. While checking the content, ask whether descriptions of original findings were referenced accordingly, as opposed to being incorrectly attributed to reviews [23].

### Consider how studies were critically evaluated

Beyond correct data summary, narrative literature reviews should include critical data appraisal and some level of data synthesis. How this should be done varies according to the review scope and methodology [9, 10, 19]. While some narrative reviews reasonably focus on breadth as opposed to depth of literature coverage [10], limited or poor data appraisal risks placing undue emphasis on poor quality research [9]. Evaluating at least some aspects of the methods used by individual studies can improve reliability [7]. Similarly, ask how the authors have interpreted conflicting findings or studies with apparently outlying results [9, 11].

### Evaluate whether tables/figures/diagrams support the text

While not all literature reviews need to include figures or tables, these can help to summarise findings and make key messages clearer. Some detailed information may be best presented in tables, with a shorter summary within the text. Tables can improve the availability of quantitative data for cross-checking, better demonstrate the results of qualitative or quantitative data synthesis, and reassure both peer reviewers and readers that comprehensive, objective analyses have been performed. If figures or tables are included, these need to be original; otherwise, the authors need to have obtained permission to reproduce these from an original source.

### Consider whether the review will help someone entering the field

Literature reviews are not always read by subject experts, and it is important that the peer-review process considers this. Reviewers who are not direct content experts may valuably request clarification of nomenclature and/or historical issues that may have seemed too obvious for the authors to have explained. Summary diagrams suggested by peer reviewers may help make a literature review more accessible to a broader audience.

### Ask whether the review expands the body of knowledge

Ultimately, the goal of a literature review should be to further the body of knowledge [18]. Extending or developing ideas is clearly a difficult task, and is often the weakest section of a review [25]. Consider therefore whether the authors have derived and clearly presented new ideas and/or new research directions from any identified knowledge gaps. Having read the manuscript with fresh eyes, peer reviewers may have valuable ideas to contribute.

### Do not forget the rules for reviewing manuscripts in general

The review of literature reviews has some particular considerations, but all the usual manuscript review rules also apply, such as managing conflicts of interest and allocating appropriate time [16, 17]. Try to separate the assessment of language and grammar from the more important assessment of scientific quality and remain aware that expert reviewers risk bringing their own biases to the peer-review process [15].

## Conclusions

More quality peer reviewers are needed within the scientific community [3], including those with the capacity and confidence to review narrative literature reviews. Although it has been difficult to identify predictors of peer-reviewer performance and effective training methods, younger reviewer age has been reproducibly associated with better quality manuscript reviews [26, 27]. This association suggests that peer reviewers should be recruited relatively early in their careers, and encouraged to participate widely in manuscript review. Associations between younger peer-reviewer age and better manuscript reviews may also highlight the need for regular training, to ensure that the peer-review community remains up-to-date regarding new approaches to editing or reviewing manuscripts. Indeed, a recent industry survey reported that over three quarters of researchers were interested in further reviewer training [28]. I therefore hope that this article will add to existing resources [29] to encourage less experienced peer reviewers to extend their efforts towards narrative literature reviews.
